# Role of microRNAs in B-Cell Compartment: Development, Proliferation and Hematological Diseases

**DOI:** 10.3390/biomedicines10082004

**Published:** 2022-08-18

**Authors:** Olívia Fonseca Souza, Ana Flavia Popi

**Affiliations:** Disciplina de Imunologia, Departamento de Microbiologia, Imunologia e Parasitologia, Universidade Federal de São Paulo, São Paulo 04039-032, Brazil

**Keywords:** microRNAs, B-cell, hematological B malignancies, exosomes

## Abstract

B-cell development is a very orchestrated pathway that involves several molecules, such as transcription factors, cytokines, microRNAs, and also different cells. All these components maintain the ideal microenvironment and control B-cell differentiation. MicroRNAs are small non-coding RNAs that bind to target mRNA to control gene expression. These molecules could circulate in the body in a free form, protein-bounded, or encapsulated into extracellular vesicles, such as exosomes. The comprehension of the role of microRNAs in the B-cell development was possible based on microRNA profile of each B-cell stage and functional studies. Herein, we report the knowledge about microRNAs in the B-cell the differentiation, proliferation, and also in hematological malignancies.

## 1. Introduction

Several molecules take part in regulating cell development, differentiation, metabolism, proliferation, and survival. Beyond that, microRNAs (miRNA or miRs) are highly conserved molecules across species [1,2] that play a central role by targeting messenger ribonucleic acid (mRNA) related to these processes. More than 30% of all human genes (protein-coding genome) are targets of miRNAs, and one miRNA molecule could have multiple mRNA targets [3,4]. Similarly, every mRNA can be regulated by more than one microRNA [5]. Biogenesis of these non-coding RNAs happens in the nucleus and cytoplasm, with the participation of different enzymes. Mouse model experiments with Dicer modification, a central molecule in this pathway, show an impaired synthesis of microRNAs and consequently disrupt the B-cell development (arrest at the pro-B to pre-B transition). Furthermore, these mice also showed other effects on cell survival and antibody diversification, showing its relevance in homeostasis maintenance of hematopoiesis and cell function [6].

Monticelli et al. [7] performed a detailed miRNA expression profile study in murine immune cell subsets. These authors reveal that the expression of miRNAs in the hematopoietic system changes depending on the differentiation status, which indicates that microRNA regulates lineage commitment in immune cells. This subject was confirmed by microarray analysis [8,9] and also reviewed by Metha et al., 2016 [10].

Based on that, any alterations in the miRNA regulation system could lead to defects in cell number and activity, as well as promote the appearance of diseases. In this review, we focus on the role of microRNAs in controlling B-1 and B-2 development and in the hematological B-cell malignancies that could appear due to disbalance in miRNAs expression.

## 2. microRNAs: Biogenesis, Activity and Function

MicroRNAs (miRNAs or miRs) are small non-coding RNA molecules, single-stranded with 19–23 nucleotides in length. They have origin in intergenic miRNA genes (canonical pathway) or encoded in introns with hairpin-forming potential (non-canonical pathway) [11]. RNA polymerase II or RNA polymerase III transcripts the miRNA genes in the canonical pathway, leading to molecules called primary miRNAs (or pri-miRNAs), that could have the 5′-cap and 3′-polyadenylated tails [12]. Pri-miRNAs are then processed and cleaved into precursor-miRNAs (pre-miRNAs) by a protein complex formed with Drosha, DGCR8 (DiGeorge syndrome critical region gene 8) and RNase III enzymes. In this process, intermediary molecules with approximately 70 nucleotides were found [13], which are transported to the cytoplasm by Exportin 5 [14]. RNase enzyme Dicer and TAR RNA-binding protein (TRBP) act on this complex and generate a miRNA-duplex with ~22 bp length [15]. Mature miRNAs can emerge from both strands: miRNA guide can be either 3′ or 5′ of the precursor stem-loop structure [16] and, usually, the passenger strand is degraded. RNA-induced silencing complex (RISC) is a complex formed by the mature microRNAs and Argonaute proteins (Ago). RISC represents a form of microRNAs’ action, since it is important to regulate gene expression by targeting specific mRNAs through partially or fully complementary base pairing in the 3′ untranslated region (UTR). This could result in mRNA degradation and/or translational repression [1,17]. In this gene silencing system, GW182 proteins also interact with Ago proteins [18,19,20]. Otherwise, miRNAs arise from splicing and formation of mirtrons in the non-canonical pathway [21,22] but generate the same miRISC complex at the end.

Since microRNAs regulate different genes involved in cell proliferation survival and differentiation, studies about these molecules could contribute to understanding the origin and prognosis of deregulations that lead to malignancies, such as solid or hematological cancers. Several studies have emerged on this area, and it is established that miRNAs in cancer are specifically called as oncomiRs, which are upregulated and contribute to tumor-promoting function, or they are named as tumor suppressor miRs, the other ones that are downregulated, interfering in the tumor suppressive function [23].

## 3. B-Cells: Populations, Location and Functions

B lymphocytes can be divided into two subtypes named as B-1 and B-2 cells. This nomenclature is defined by the time of appearance during ontogeny.

B-2 cells are known as the conventional B lymphocytes, which participate in adaptive immune response, are antigen-presenting cells and mediate humoral immunity by secreting high-affinity antibodies. These cells can generate immunological memory. B-2 cells are defined with CD5^-^CD19^+^CD23^+^B220^+^IgM^low^IgD^high^CD43^-^ phenotype [24]. These cells differ from B-1 cells that mediate innate immune response, secreting natural antibodies and participating in the first line of defense against a variety of pathogens, as reviewed by Baumgarth, 2019 [25].

The discovery of B-1 cells is more recent, and these cells were early identified as “Ly-1 B-cells” based on the expression of surface antigen Ly-1 in mice or Leu-1 in humans, nowadays called CD5 [26]. The knowledge about this subtype is increasing with time, but a lot remains to be elucidated, principally the differences in B-1 cell characteristics between mice and humans. In mice, B-1 cells have CD19^high^CD23^-^B220^low^IgM^high^IgD^low^CD43^+^ phenotype [24,27,28] and in humans, CD20^+^CD27^+^CD43^+^CD70^+^ [29]. Despite this, these cells have similar features, such as CD5 expression, natural antibody secretion, allogenic T cells proliferation induction, and presence of tonic signaling in the absence of direct B-cell receptor engagement [28]. The discovery of B-1 cells was related to the expression of CD5 in B-cells from chronic lymphocytic leukemia (CLL) [30] and other tumors [31], revealing the relation between these cells and hyperproliferative diseases. These cells are also related to autoimmune diseases [32], since these cells secrete immunoglobulin spontaneously without antigen interaction [33]. It is relevant to mention that CD5+ B-cells were originally described in an autoimmune mouse strain, the New Zealand Black (NZB) [34].

## 4. B-2 Cell Development

B-cell potential is present in embryonic life, but the major contributor to B-2 development is the adult bone marrow, an organ that has the ideal niche that provides support for self-renewal and differentiation of HSCs into mature blood cells [35]. All processes described below occur in mammals.

The pathway initiates with the asymmetric division of hematopoietic stem cells (HSCs) in multipotent progenitors (MPP). These cells can differentiate in granulocyte multipotent progenitors (GMP)—these give origin to macrophages and neutrophils—, in megakaryocyte–erythroid progenitors (MEP)—where megakaryocytes and erythrocytes originate—or in lymphoid-primed multipotent progenitors, with expression of E2A, Ikaros and PU.1 transcription factors. Diminution in PU.1 expression but maintenance of Ikaros and E2A leads to differentiation in a common lymphoid progenitor (CLP). This cell has two distinct pathways: generating T cells from early T-cell progenitors (ETP) together with the expression of E2A and HEB or B-cells from B-cell-biased lymphoid progenitors (BLP). The differentiation in BLP happens with the expression of E2A and HEB, which activates the expression of Foxo-1, and to generate pro-B-cells, Foxo-1 acts together with E2A to activate EBF1. In this stage, EBF1, along with E2A, binds to the *Igh* locus to start D_H_J_H_ recombination in the immunoglobulin rearrangement process. By a positive intergenic feedback circuitry, EBF-1 and FOXO1 activate the B-lineage specific program, which involves PAX5 expression, contributing to the commitment to the B-cell fate and generation of pro-B-cells [36,37,38,39,40,41]. PAX-5 is also important to promote V_H_ to D_H_ recombination, indicating that the cells are in the late-pro-B stage. In the early pre-B-cell stage, cells had completed V_H_ to D_H_J_H_ rearrangement. After the successful recombination, B-cells express a pre-BCR, and only cells with a productively rearranged heavy chain are selected, indicating the first checkpoint of the B-cell development, or positive selection. The next stage is the late pre-B-cell, in which light chain rearrangement mediated by *Rag1/2* gene expression occurs, which culminates in IgM receptor integration to generate BCR on the cell surface. B-cells can undergo negative selection or receptor editing if self-reactivity is detected. After this, immature B-cells exit bone marrow to complete maturation [42,43], and the principal location of B-2 cells is the secondary lymphoid organs, such as the spleen and lymph nodes. In the periphery, the B-cell development is antigen-dependent, and cells differentiate into T1 and T2 transitional cells until they become mature B-cells following BCR-mediated signals and germinal-center reactions (GC reactions). Importantly, B-cells undergo Ig affinity maturation by somatic hypermutation and in germinal center reactions, Ig genes undergo class switch recombination, both steps dependent on AID (activation-induced cytidine deaminase) activity. Mature cells in the periphery are follicular B-cells (FOB) or marginal zone B-cells (MZB), depending on different signals: BCR signaling pathways integrated with BAFFR and Notch2 or migration/retention signals [44,45]. After leaving germinal centers, B-cells are memory B-cells or plasmablasts, and this process is regulated by activation of BLIMP-1 and XBP1 [46].

## 5. B-1 Cell Development

B-1 cells are the other subtype of B-cells and two distinct subpopulations are known and characterized by the CD5 expression: B-1a cells express this marker, whereas B-1b cells do not [47]. The discovery of CD5+ cells in health and disease lead to the investigation of B-1 cell origin and development in mice. The difference in B-1 and B-2 progenitors was revealed in studies with various donor tissues: neonatal liver efficiently reconstituted B-1 cells while adult bone marrow majorly reconstituted B-2 cells counterpart, after transfer into irradiated mice [48,49]. This also confirmed the hypothesis of Herzenbergs that “conventional B and B-1 cells belong to separated lineages deriving from distinct progenitors emerging at different times during development” [50], as early B-1 progenitors were found in multiple fetal tissues, such as embryonic yolk sac and para-aortic splanchnopleura around day 9–9.5 of gestation [51]. This suggested the B-1 cells’ emergence in waves, starting in the yolk sac from nonhematopoietic precursors [52,53,54], then in the aorta-gonad-mesonephric region [55], fetal liver, and only starts on day 15 of gestation in bone marrow [56]. This also revealed the B-1 cells’ origin from precursors independent or dependent on hematopoietic stem cells (HSCs), since HSCs only appear after day 10 [35,57], bringing the idea that at least two distinct progenitors contribute to B-1 cell appearance, as shown by Beaudin et al. [58].

Using Hardy scheme [59], B-1 cell specific progenitors were identified with Lin^−^CD93(AA4.1)^+^CD45R^−/low^CD19^+^ phenotype, in higher frequency in fetal liver on day 17 of gestation [60]. Early B-2 progenitors expressed CD45R but lacked CD19 in this study, confirming the lineage model that proposed distinct progenitors for B-cells.

These B-1 cell progenitors represent pro-B stage and the development pathway follows up the same steps of B-2 cell differentiation and maturation (from pro-B-cells to mature cells), although Ikaros and PAX-5 do not constrain the expression of PU.1 in B-1 cell development [61]. Additionally, B-1 cells’ repertoire reflects the development; these cells are positively selected based on self-antigens binding [62], and differences between B-1 and B-2 were observed in relation to pre-BCR signaling in pro or pre-B-cells [63]. Other observed differences rely on antigen influence for B-1 cell development and expansion; these cells do not need antigen participation to be activated and secrete IgM, whereas B-2 cells are selected, activated and enter in clonal expansion after antigen binding in the immunoglobulin named BCR, receptor already mentioned above.

## 6. microRNAs in the Early B-Cell Development

Different microRNAs were described as positive or negative regulators of early B-cell development. Beyond them, there is miR-17-92 cluster, miR-34a, miR125b, miR-150, miR-23a cluster, miR-181a and miR-212/132 cluster. All contributions are detailed as described below. In this section, we also cite microRNA regulation impact on processes such as cell differentiation, migration, invasion and cell cycle that could culminate in a hematological disease. Cancers affecting B lymphocytes can arise from progenitors or mature B-cells and in the next sections, we define the most common B-lymphomas and discuss the proliferation of mature B-cells and microRNAs contribution to that.

As mentioned before, one decisive step in B-cell development is the Ig rearrangement, which indicates the cell stage. In this process, several microRNAs regulate pro-B to pre-B-cell transition. Mice deficient in miR-17-92 cluster (e.g., Mb1tKO) [64,65] presented B-cell development inhibited at the pro-B stage, but when these cells were transfected with lentiviral vector expressing miR-17, the transition to pre-B was rescued [65]. Based on that, it is conclusive that this cluster, composed by six mature miRNAs (miR-17, -18a, -19a, -20a, -19b-1 and -92a-1) and two paralogs (miR-160a~363 and miR-106b~25) [66], is important to generate B-cells, highlighting miR-17 participation. More studies are needed to understand the relation of other miRNAs in the cluster and the stage development. Some studies tried to indicate by which target miR-17 regulates the development and identified the pro-apoptotic BIM (Bcl-2-like protein 11), indicating that the *Bim* targeting leads to survival of B-cell progenitors [64]. In silico analysis also revealed that *Bim* 3′-UTR has several sites that closely match the seed sequence of 17~92. However, expression of BIM in pro-B-cells from study with Mb1tKO contradicted this [65], suggesting other targets to this miRNA cluster. Another validated target is the transcription factor E2 family [67].

Expression of miR-17~92 cluster has been related to microRNA control in the early steps of lymphocytes development, since it is a major cluster with a role in B and T differentiation and diseases [68,69]. It is known that miR-17-92 cluster is involved in autoimmunity [65,70] because miR-19 inhibits phosphate and tensin homolog (PTEN) expression, which negatively regulates PI3K-AKT pathway, leading to a hyperactivation of self-reactive B-cells. Additionally, this cluster showed an oncogenic potential by regulation of c-myc, specially seen in lymphomas [71]. Along with this, MIR17HG amplification is present in patients with large B-cell lymphoma, and miR-18a expression is associated with prognosis and patients’ survival [72]. Moreover, miR-17 and -19b-1 are overexpressed in B-cell culture from CLL patients, and miR-20a is now studied as a biomarker of this disease [73,74]. In multiple myeloma (MM), miR-17 is also oncogenic and affects iron transport by regulating Ferroportin 1 (FPN1), in which decreased FPN1 levels leads to MM cell growth and proliferation and also correlates with bad prognosis [75]. Finally, the paralogue miR-106b acts in loop with an important regulator in carcinogenesis, the Kruppel-life factor 10 (KLF10) [76].

Other miRNA involved in the pro-B to pre-B-cell transition is miR-181, which is naturally increased in tissues such as bone marrow, thymus and spleen. miR-181 overexpression studies revealed increased B-cell lineage in vitro and in vivo, in which lineage negative bone marrow cells (Lin) differentiates to B-cell lineage [77]. However, the absence of miR-181 leads to a modest impairment in B-cell development [78]. Differently from miR-17-92, miR-181 acts as tumor suppressor and inhibits cell proliferation due to NF-kB regulation, as seen in studies with DLBCL xenograft. Direct targets of miR-181 involved in NF-kB repression are the transcription factor p65 (RELA), protooncogene *c-Rel* (CREL) and *p50* gene [79]. However, miR-181 could act as oncomiR by interfering in DLBCL progression cells by repressing *CARD11* [80]. In multiple myeloma, miR-181a is upregulated and has a target in *CCND1*, a cell cycle regulatory gene. The downregulation of this miRNA inhibited expression of *CCND1* and also caused cell cycle arrest [81]. A recent meta-analysis showed that upregulated expression of miR-181 predicts favorable outcomes of patients with cytogenetically normal acute myeloid leukemia and chronic lymphocytic leukemia [82]. Interestingly, miR-181b is down-regulated in CLL patients, and this is related to poor prognosis, but more studies are necessary to investigate these targets [83].

miR-34a is also related with pro-B to pre-B transition, since studies with transduction of bone marrow cells with a retroviral expressing miR-34 revealed blockage at pro-B stage and reduced mature B-cell numbers. The regulation happens due *Foxp1* suppression, which is important to this step [84]. In Acute Myeloid Leukemia (AML), miR-34 acts as a tumor suppressor gene by targeting high motility group box 1 (HMGB1), a DNA-binding protein that can repair DNA mismatch and regulate gene transcriptions [85]. In preclinical in vivo models for MM several targets were identified to microRNA-34, such as *BCL-2*, *CDK6*, *Cyclin D*, *SIRT1* and *VEGF* [86] and *RMRP* [87].

miR-150 is increased in immature B-cells and is important to regulate *Myb* and *Foxp1*, important molecules in pro-B-to-pre-B transition. Bone marrow transplantation with HSCs overexpressing miR-150 revealed defects in mature B-cell numbers and blockage at pro-B stage [88,89]. This miR is involved in lymphoma and melanoma. In follicular lymphoma, it was seen that regulation by *MYC* and downregulation of miR-150 contribute to high-grade transformation due to the upregulation of Foxp1, which is a positive regulator of cell survival [90]. In melanoma, a study recently described cell proliferation, migration and invasion suppression by targeting the 3′-UTR region of Sine oculis homeobox 1 (*SIX1*) [91], gene known in the tumorigenesis process of several cancers and also by downregulation of *MYB* [92]. Conversely, restoration of *MYB* in miR-150-overexpressed melanoma cells rescued the proliferation, migration, and invasion [92].

miR-23a, miR-24-2, and miR-27a form the miR-23a cluster that is a downstream target of PU.1 transcription factor. Expression of this cluster leads to differentiation in myeloid progenitors, simulating PU.1 action. Additionally, deficient mice in all three miRNAs showed increased numbers of B lymphocytes in bone marrow and spleen, in comparison with myeloid cells [93,94]. Similarly, overexpression of the whole cluster in hematopoietic stem progenitor cells (HSPCs) repressed CD19-positive B development [93]. Expression of this miRNA was correlated with diffuse large B-cell lymphoma, which promotes proliferation, invasion and metastasis through the target inhibition of metastasis suppressor 1 *(MTSS1*) [95]. The expression of miR-27 also enhanced the proliferation, migration and invasion of MM cells by targeting *SPRY2* [96].

Ultimately, the last miRNA cited as a known regulator of early B lymphocyte development is the miR-212/132 cluster. Inhibition of development in pre-pro-B to pro-B-cells was observed when mice overexpressed miR-132, resulting in the failure in transcription factor Sox4 regulation [97], and these mice did not present any B-cell phenotype. Sox4 is important to *Rag-1* expression and deregulation in this axis leads to defects in VDJ recombination. The development in these mice could be rescued by the expression of a SOX4 mutant without miR-132 binding site. The only hematological disease related to this cluster is CLL, where miR-132 is increased [98], but further patients studies are needed.

**Figure 1:**
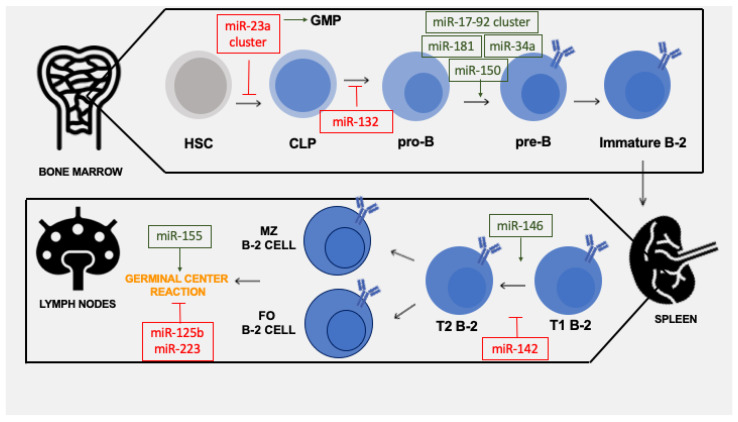
MicroRNAs participate in the B-cell differentiation and maturation by negative control (red) or induction (green) of genes related to B-cell counterparts. In bone marrow stages, microRNAs miR-17-92 cluster, miR-181, miR-34a, and miR-150 act along cell differentiation. Contrariwise, miR-23a and miR-132 blocks B cell differentiation. In the periphery, miR-146 and miR-142 have roles in transitional B-cells in the spleen and miR-155, miR-125 and miR-223 regulate germinal center reaction in lymph nodes.

The information here revealed that several microRNAs regulate B-cell development and any defects on this path could lead to hematological diseases. Figure 1 describes microRNAs that positively (green) or negatively (red) regulates B-cell development and differentiation.

## 7. microRNAs in the Early B-1 Cell Development

A lot remains unelucidated about B-1 cells, and it includes miRNAs participation in cell development and growth. However, some studies showed the involvement of let-7 family.

Let-7 miRNA family was discovered in *Caenorhabditis elegans*, which are regulated by LIN28b and have as target the transcription factor ARID3a. Pro-B-cells or HSPCs from mice bone marrow expressing LIN28b are more prone to giving rise to B-1a cells than B-2. Ectopic let-7 expression in pro-B-cells from murine fetal liver gives rise to B-2 cells at the expense of B-1a cells. Additionally, when pro-B-cells from adult mice bone marrow overexpressing ARID3a were transferred to immunodeficient mice, there is an increase in B-1 cell differentiation. Fetal pro-B-cells deficient in *Arid3a* (knockdown with short hairpin RNA) led to reduced B-1a development. All this data indicates the loop involving B-cell differentiation and let-7 participation (reviewed by [42]).

**Figure 2:**
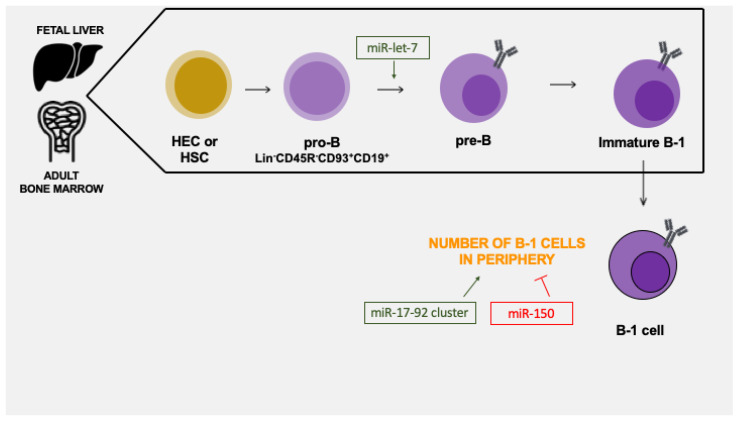
Role of microRNAs in B-1 cell differentiation. A few microRNAs are known to regulate B-1 cell stages by negative control (red) or induction (green). In bone marrow, let-7 interferes in pro-B-to-pre-B differentiation. In the periphery, microRNAs miR-17-92 cluster and miR-150 control B-1 cell quantity.

Figure 2 describes microRNAs that positively (green) or negatively (red) regulate B-1 cell development and differentiation. Complementary studies with adoptive bone marrow transplantation are needed to understand the role of miRNA in B-1 cell progenitors, as well as studies with fetal progenitors to elucidate miRNAs in early B-1 cell life.

## 8. miRNAs Regulate B-1 Cell Numbers in the Periphery

Not enough knowledge of miRNA influences in mature B-1 cells exists. However, miR-150 and miR-17-92 cluster revealed a role in regulating the number of peripheral B-1 cells. miR-150 negatively regulates B-1a cell development due the upregulation of MYB. This was proved in miR-150 knockout mice that have increased B-1 cell numbers in the spleen and peritoneal cavity. Additionally, ectopic expression of this miRNA, as well as deletion of one allele of *Myb*, result in reduced B-1 cell counterpart [88].

B-1a cell numbers are reduced in mice with deletion of miR-17-92 by Cre in CD19 cells. Additionally, it was seen that B-1a cells need CD19 expression to develop via activation of PI3K signaling pathway and miR-17-92 overexpression can rescue B-1a development in the peritoneal cavity of mice deficient in CD19 as a consequence of PI3K activation [99,100]. Thus, more studies are needed to identify microRNAs as central regulators of B-1 cell quantity.

## 9. miRNAs Involvement in Regulating Periphery B-Cells

In order to understand microRNAs influence on immature B-cells differentiation to T1, T2, MZB or FOB cells and also in GC response, Ig isotype switching and somatic hypermutation, several studies investigated the quantity of B-cells in the periphery as well antibody production, after alteration in miRNA expression.

miRNAs such as -146a, -155, -125b, -223 and -142 revealed a role in regulating B-cell maturation, by targeting transcription factors involved in antigen-dependent B-cell differentiation. However, to better understand microRNA participation in the periphery, profiling during B-cell activation still needs investigation such as in silico prediction of targets involved and functional studies.

Deficient mice in miR-146 presented diminished numbers of MZB cells in the spleen while T1 and T2 cells were increased, showing the role of miR-146a in peripheral B-cell differentiation. The mechanism involved is the target of *Numb*, which causes inhibition of the Notch2 pathway [101]. Mice deficient in miR-155 have reduced GC reaction and deficient secretion of class-switched high-affinity antibodies, since this microRNA negatively regulates AID [102]. Studies presented GC B-cells number reduced when B-cells lacked miR-155 expression, whereas this condition was reverted when overexpressing miR-155, in which mice presented increased GC B numbers and also enhanced antibody response [103]. Recently, a new target of miR-155 called DEPTOR was described. DEPTOR is an mTOR phosphatase that controls in migration and cytokine production of cells from DLBCL mediated by BCR signaling [104]. In another disease, CLL, it was seen that patients presented increased expression of miR-155, and this was correlated with poor prognosis [105]. Considering this scenario, a recent NGS study shows the miR-155 role in the regulation of cell cycle phases and checkpoints. miR-155 is differentially expressed in CLL cells in phases G0, G1, S and G2. In G0, it regulates Myc, p53 and CDK4; in G1, it regulates CDK6 and CDK2; in S phase, it regulates CDK2; in G2 phase, it regulates WEE1/CDK1/2 and PLK1 [106]. However, patients with MM have downregulation of miR-155, which is different from the other diseases mentioned and miR-155 inhibitor is not a valid therapeutic approach [107].

Another microRNA that regulates GC reactions and IgM production is miR-125b, by targeting IRF-4 and BLIMP-1 transcription factors. In vitro studies showed that B-cells overexpressing miR-125b mimic and stimulated with LPS had reduced differentiation into plasma cells and IgM production [108]. miR-223 is upregulated in naïve and memory cells in comparison to GC cells, also revealing a role in this reaction. The probable targets by which this regulation occurs are the transcription factors LMO2 and MYBL1 [109]. In MM cells, miR-125b is increased, and inhibition of it reduces AKT levels, showing other potential targets to diminish proliferation. Another direct target is PHLPP2 (Pleckstrin homology domain leucine-rich repeat protein phosphatase), which acts as a negative regulator of the PI3K pathway. This shows that miR-125b is also important to control B-cell activation/proliferation [110].

miR-142 can be related to hypoimmunoglobulinemia and immunoproliferative disorder. miR-142 gene deletion resulted in enlarged MZB population, whereas B-1 cells were decreased and IgM reduced [111].

**Figure 3:**
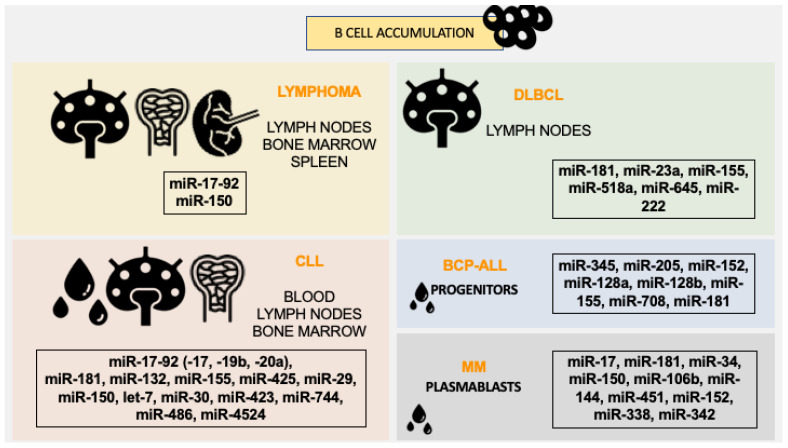
OncomiRs related to B-cell accumulation and proliferation in hematological B malignancies: Lymphoma, DLBCL, CLL, BCP-ALL and MM. Lymphoma disease is characterized by the accumulation of cells in lymph nodes, bone marrow and spleen. MicroRNAs miR-17-92 and miR-150 are known to regulate cell numbers in these organs. DLBCL (Diffuse large B-cell lymphoma) present B-cell number increased in lymph nodes, and microRNAs miR-181, miR-23a, miR-155, miR-518a, miR-645 and miR-222 can interfere in this accumulation. CLL (Chronic Lymphocytic Leukemia) has a higher number of B-cells in blood, lymph nodes and bone marrow, where miR-17-92 (-17, -19b, -20a), miR-181, miR-132, miR-155, miR-425, miR-29, miR-150, let-7, miR-30, miR-423, miR-744, miR-486 and miR-4524 showed a role in control cell accumulation and proliferation in these tissues. BCP-ALL (B-Cell Precursor Acute Lymphoblastic Leukemia) is the accumulation of B-cell progenitors in blood and microRNAs miR-345, miR-205, miR-152, miR-128a, miR-128b, miR-155, miR-708 and miR-181 are characterized as biomarkers of B-cell number control. Lastly, MM (Multiple myeloma) where plasma blasts accumulate in the blood, has microRNAs miR-17, miR-181, miR-34, miR-150, miR-106b, miR-144, miR-451, miR-152, miR-338, miR-342 as regulators of proliferation and accumulation.

Other miRNAs, despite the described in B-cell development, also have a role in B-cell proliferation and are related to hematological diseases. The most common B-lymphomas are characterized here. Its oncomiRs that impact proliferation are discussed below and summarized in Figure 3.

Diffuse large-B-cell lymphoma (DLBCL) occurs mainly in older people, but can affect people of any age, and it starts with a growing mass in a lymph node. Another disease that happens mostly in elderly people is Chronic Lymphocytic Leukemia (CLL), where the accumulation of cells happens in blood and bone marrow. The Small Lymphocytic Lymphoma (SLL) is often considered a different version of CLL, where cells accumulate in lymph nodes and spleen. Both are slow-growing (indolent) malignancies in most cases. The slow-growing Follicular Lymphoma is very rare in young people, and the average age of patients is equal to 60 years. It happens in many lymph node sites in the body and in the bone marrow. The Mantle Cell Lymphoma (MCL) is also known to appear in people older than 60 years but is much more common in men than in women. It is diagnosed with a widespread disease in the lymph nodes, bone marrow and spleen, and the tumor tends to grow fast. The disease from plasmablasts, the last step of B-cell differentiation, is called multiple myeloma (MM). From precursor B-cells, there is the B lymphoblastic leukemia/lymphoma, a disease characterized by an increase in bone marrow blasts [112,113].

Lymphoma tissues and cell lines show a decrease in miR-553 levels. This microRNA is involved in proliferation, as described by a recent study in which overexpression inhibited proliferation and promoted apoptosis. Moreover, ß-catenin was identified as a target to this miRNA, with bioinformatics prediction and also with luciferase assays [114].

The relation of miR-345 in the pathogenesis of B-cell precursor acute lymphoblastic leukemia (BCP-ALL) was seen, where its overexpression suppressed cell proliferation and boosted apoptosis of NALM-6 and RS4;1 cells [115]. Still in BCP-ALL, expression of miR-205 was found downregulated in patients, and overexpression restrained proliferation and promoted apoptosis of the cells. In this study, MALAT1, a long non-coding RNA (lncRNA) was identified as a regulator of ALL cell proliferation by regulating miR-205. This last has a target in PTK7, a protein tyrosine-kinase [116]. miR-152 is also regulated by a lncRNA (LINC00221) and presents a role in ALL cell proliferation and apoptosis [117]. Additionally, a recent review showed that miR-128a, -128b, 155, -708 and -181 family were related to B-ALL, where they were all upregulated [118].

In DLBCL, a recent study showed the role of miR-518a-CCR6 loop, indicating that this feedback regulates proliferation [119]. Moreover, miR-645, upregulated in DLBCL tissues and cell lines, is involved in proliferation. Its downregulation inhibited proliferation, cell cycle progression and promoted apoptosis of cells by targeting Dachshund family transcription factor 1 (*DACH1*), bounding in 3′-UTR [120]. Another microRNA that inhibits the proliferation of DLBCL cells is miR-196a-3p, which is downregulated in this disease and targets ADP ribosylation factor 4 (ARF4) and also promotes cell apoptosis [121]. In contrast, overexpression of miR-222 promoted proliferation and inhibited apoptosis of DLBCL cells, by targeting Phosphatase 2 regulatory subunit B alpha (PPP2R2A) [122].

In CLL, it was described that miRNA-425 inhibits CLL cell proliferation by a mechanism involving BTK (Bruton tyrosine kinase) [123]. miR-29a, miR-150 and miR-155 were upregulated in the early stages of CLL but were modest predictive biomarkers of CLL risk [124]. Other microRNAs such as let-7e, miR-30, miR-423, miR-744, miR-486, and miR-4524 were found differentially expressed in CLL patients and have a role in the regulation of cell cycle phases and checkpoints, revealing its participation in proliferation once more. These microRNAs regulate cyclins and cyclin-dependent kinases, e.g., Cyclin A, B, D, and E, and CDKs 1, 2, 4 and 6. Moreover, they also regulate other gene expressions such as *MYC*, *P53*, *ATM*, *PLK1*, *CDC25A*, and transcription factors ATR and E2F [106].

Similarly to other diseases described above, MCL also has a relation with miR-17-92 cluster, which is upregulated in MCL cells and modifies cell proliferation [125]. The cluster miR-106a~363, which is composed of microRNAs -18b, -19b-2, -20b, -92a-2, -106a, and -363, appears to function in cell proliferation in the same way as paralogous miR-17-92 cluster, but this remains to be elucidated [126]. Moreover, miR-100 inhibits cell proliferation by targeting mTOR [127], miR-15a/16-1 cluster is downregulated in MCL patients and interferes in cell cycle regulation by CCND1, which is a direct target of these two microRNAs [128] and the miR-29 family also have participation in regulating MCL cell proliferation, regulating cyclins expression [129,130,131].

Multiple miRNAs are related to multiple myeloma. miR-144, downregulated in MM patients and MM cell lines, targets MEF2a (myocyte enhancer factor 2A) and inhibits the proliferation, migration and angiogenesis of MM cells and the overexpression reverses the effects [132]. The direct target of miR-144 is transmembrane tyrosine kinase c-MET, which is involved in invasion, migration, proliferation and angiogenesis [133]. miR-144 also induced apoptosis in a recent study by inhibiting Wnt/ß-catenin pathway [134], and similarly, miR-451 induced cell death by targeting c-Myc [135]. Inhibition of miR-338 action, upregulated expression of BRD4, and augmented MM cells proliferation [136]. Another study showed the activation of BRD4 signaling via targeting miR-152 in MM cells [137]. Overexpression of miR-342 in MM cells suppressed cell proliferation, arrested cell cycle transition from G1 to S phase, and promoted apoptosis of MM cells [138]. Moreover, miR-26a diminished cell proliferation and migration by targeting CD38 [139]. Other microRNAs related to inhibition of MM cell proliferation are miR-25, which targets the PTEN/PI3K/AKT signaling pathway [140]; miR-29b, by targeting DNMT3A/B and HDC4 [141,142]; miR-30-5p, which targets BCL9 [143]; miR-192 by suppressing TGIF2 [144] miR-140-5p, which targets VEGFA [145] and miR-489 by targeting LDHA [146]. All these data reveal the importance of microRNAs in controlling cell proliferation and accumulation.

## 10. miRNAs Derived from Exosomes

Another important fact to be considered is that microRNAs are released from the production site in the free or protein-bounded form and can also be encountered in circulating extracellular vesicles or exosomes derived from tumor cells. Exosomes are products from the endosomal sorting pathway consisting of a bilayer membrane with 20–150 nm that can transport proteins, nucleic acids, lipids, metabolites, and organelles, which can contribute to tumor metastasis, angiogenesis, drug resistance, and affect other biological process. Importantly, exosomes contain specific constituents but also carry components that reflect the cell of origin [147,148]. Exosomal microRNAs stay protected from RNase of biological liquids and can be present in many body fluids, revealing an important molecule that can affect lymphomagenesis, for example, and also represent a biomarker in early detection and prognosis of hematological malignancies [149]. Moreover, the detection of exosomal miRNAs replaces some invasive methods (bone marrow aspiration) and dependence on leukemic cells in the blood, for example. Exosomes are detectable in systemic circulation to provide a lot of useful information for prediction [150].

Cells from hematological malignancies, like other tumors, produce exosomes. These products have a role in promoting the progression of cancer, are implicated in reprogramming of the bone marrow microenvironment, could promote drug resistance and carry oncogenic proteins [151]. Thus, microRNAs, which control cell differentiation, maturation and proliferation processes could be derived from exosomes originating in hematological malignancies and can reveal important biomarkers.

In CLL, it was described that an increased amount of miR-150 and miR-155 in exosome cargo [152] and miR-146a and -148a seem to have a relation in this disease too [153]. Additionally, miR-155 has been found downregulated in exosomes from patients with multiple myeloma [154] and lymphoma [155]. Other miRNAs identified in MM exosomes include miR-135b, let-7b and miR-18 [156]. As we reviewed, the microRNAs from the let-7 family are very important in the development of B-cells. This family is also present in the exosome cargo. In Hodgkin lymphomas, let-7 family was found in exosomes [157], and in CLL, let-7g was also described. In DLBCL, decreased exosomal miR-107, decreased miR-375-3p and increased exosomal miR-385-3p have importance in diagnostics, highlighting the miR-107, which targets 14-3-3n and restrains tumorigenesis by suppressing cell proliferation and invasion [158].

Importantly, a recent study demonstrated a series of exosomal panels related to leukemias, myeloma and lymphomas, illustrating the already mentioned microRNAs in this review, such as miR-150, -155, -1246, -17-5p, -20a-5p, -16-5p, -15a-5p, -192-5p, -21-5p, -320b and let-7d [150]. This shows the importance of studying exosomes and other extracellular vesicles as a source of microRNAs to become biomarkers or targets for treatments.

## 11. Concluding Remarks

It is established that microRNAs have an important role in controlling several processes to maintain host homeostasis. By controlling gene expression, microRNAs participate in development, differentiation, metabolism, proliferation, and survival. Here, we show the early and the most advanced studies in B-cell differentiation and proliferation related to microRNA control. Deregulations in microRNA expression can lead to hematological diseases, such as lymphomas, leukemias, and myeloma. Additionally, we show that microRNAs can be released in a free form or in exosomes, indicating a new perspective on using microRNAs as biological targets in hematological diseases.

This review focused on B development and proliferation, showing the principal microRNAs and their targets. Several others deeply describe microRNAs expression in hematological diseases [159,160]. However, a lot remains unelucidated about some targets that microRNAs can bind. That is because a lot of studies use bioinformatic analysis to predict targets but do not pursue functional experiments to confirm. Moreover, several microRNAs are described to regulate B-2 development, but few studies show this in B-1 cell subtypes. This indicates that there is a lot to discover, and this can contribute to finding biomarkers to diagnose or treat diseases related to failures in B-cell development and/or proliferation.

## Data Availability

Not applicable.
